# Qingxuan Jiangya Decoction Mitigates Renal Interstitial Fibrosis in Spontaneously Hypertensive Rats by Regulating Transforming Growth Factor-*β*1/Smad Signaling Pathway

**DOI:** 10.1155/2017/1576328

**Published:** 2017-12-26

**Authors:** Wangyu Liu, Shan Lin, Qiaoyan Cai, Ling Zhang, Aling Shen, Youqin Chen, Jianfeng Chu, Jun Peng

**Affiliations:** ^1^Academy of Integrative Medicine, Fujian University of Traditional Chinese Medicine, 1 Qiuyang Road, Minhou Shangjie, Fuzhou, Fujian 350122, China; ^2^Fujian Key Laboratory of Integrative Medicine on Geriatrics, Fujian University of Traditional Chinese Medicine, 1 Qiuyang Road, Minhou Shangjie, Fuzhou, Fujian 350122, China; ^3^Case Western Reserve University School of Medicine, Rainbow Babies and Children's Hospital, Cleveland, OH 44106, USA; ^4^Chen Keji Academic Thought Heritage Studioc, Fujian University of Traditional Chinese Medicine, 1 Qiuyang Road, Minhou Shangjie, Fuzhou, Fujian 350122, China

## Abstract

Qingxuan Jiangya Decoction (QXJYD) is a traditional Chinese medicine commonly used in the clinical treatment of hypertension. Earlier studies had shown that QXJYD could inhibit the elevation of blood pressure in spontaneously hypertensive rats (SHRs) and prevent remodeling of arterial vessels. This study examines the therapeutic efficacy of QXJYD against elevated blood pressure using the SHR model, as well as the mechanisms behind its antihypertensive activity and protection against renal fibrosis. The results showed that QXJYD significantly attenuated the increase in blood pressure in SHRs and mitigated the development of renal interstitial fibrosis. In addition, QXJYD also robustly decreased the excess accumulation of extracellular matrix and attenuated the elevated expression of MMPs. The antihypertensive effects and renal protection of QXJYD were determined to be strongly associated with inhibition of TGF-*β*1/Smad signaling pathway.

## 1. Introduction

Hypertension is the primary risk factor for heart disease and the common cause of renal disease [1]. One of the most important complications of hypertension is renal hypertension, which is associated with a global prevalence rate as high as 42%. Moreover, approximately 18% of patients with hypertension develop chronic kidney disease (CKD), which is often characterized by renal interstitial fibrosis and leads to the functional deterioration and eventual loss of renal function [2, 3]. Renal interstitial fibrosis is characterized by tubular atrophy, interstitial leukocyte infiltration, and increased interstitial matrix deposition [4]. Progression of renal interstitial fibrosis is due to imbalances between synthesis and degradation of extracellular matrix (ECM) components, which leads to excess ECM accumulation and deposition, eventually causing widespread fibrosis [5, 6].

Transforming growth factor-*β*1 (TGF-*β*1) is one of the strongest known fibrogenic factors, which can promote the synthesis of ECM components and inhibit its degradation. TGF-*β*1 has been demonstrated to play a key role in the pathogenesis of renal interstitial fibrosis [7]. Upregulation of TGF-*β*1 signaling is widely known to be activated following chronic kidney injury [2, 3]. TGF-*β*1 functions by activating interstitial fibroblasts in order to produce a large number of ECM components and thereby inducing renal fibrosis. These actions of TGF-*β*1 eventually lead to glomerulosclerosis and tubulointerstitial fibrosis and ultimately result in end-stage renal failure [8]. During renal fibrosis, TGF-*β*1 activity is regulated by Smad-dependent signaling pathways to maintain its biological and pathological activities. In canonical TGF-*β*/Smads pathway, the binding of TGF-*β*1 to its receptor type II (TGF-*β*RII) activates the TGF-*β* receptor type I (TGF-*β*RI) kinase. Consequently, TGF-*β*RI phosphorylates Smad2 and Smad3, which bind to Smad4 to form the Smad complex. Finally, this complex is transported into the nucleus in order to regulate the gene expressions of various fibrotic markers, including Collagen I, Collagen IV, and MMPs [9–11].

QXJYD has long been used in China for the clinical treatment of hypertension. Many active components have been identified in QXJYD, such as rhynchophylline, gastrodin, and baicalin, which have been shown to possess antihypertensive activities [12–15]. However, the molecular mechanisms of the QXJYD in protecting against hypertension remain unclear. In the present study, we researched the content of QXJYD by HPLC, also used an SHR model to determine the effects of QXJYD in attenuating elevated systolic blood pressure, and examined its therapeutic efficacy against hypertension [16]. To further investigate the underlying mechanisms of QXJYD in protecting against hypertension, we evaluated its effect on renal interstitial fibrosis in an SHR model of hypertension. Therefore, this study provides a theoretical basis for the widespread use of QXJYD in the prevention and treatment of hypertension.

## 2. Materials and Methods

### 2.1. Materials and Reagents

TGF-*β*1, Smad2/3, MMP-2, MMP-9, TIMP-1, Collagen I, Collagen III, and Collagen IV primers were synthesized by Invitrogen (Grand Island, NY, USA). TRIzol reagent, PrimeScript™ RT reagent Kit, and SYBR Premix Ex Taq II Kit were provided by Takara Biotechnology Co., Ltd. (Dalian, Liaoning, China). TGF-*β*1, Smad2/3, P-Smad2/3, Smad4, MMP-2, MMP-9, GAPDH, and anti-rabbit secondary antibodies were purchased from Cell Signaling Technology (Beverly, MA, USA). All other chemicals and reagents were obtained from Sigma Chemicals (St. Louis, MO, USA).

### 2.2. Animals

20 male spontaneously hypertensive (SHR) rats (age: 4 weeks; weight: 200 g) and 10 normotensive control Wistar Kyoto (WKY) rats (age: 4 weeks; weight: 200 g) were obtained from SLAC Laboratory Animal Technology Co. Ltd. (Shanghai, China). All rats were maintained under specific pathogen-free conditions with controlled humidity, temperature (22°C), 12 h light/dark cycle, and free access to food and water. All animal experiments were performed strictly in accordance with international ethical guidelines and the National Institutes of Health Guide concerning the care and use of laboratory animals and were approved by the Institutional Animal Care and Use Committee of Fujian University of Traditional Chinese Medicine.

### 2.3. Drug Administration and Blood Pressure Measurement

Rats were acclimatized for one week prior to experiments and were divided into three groups: WKY, SHR, and QXJYD (*n* = 10 for each group). Rats in QXJYD group were given oral treatment of 60 mg/kg/day QXJYD, while rats in WKY and SHR group were treated with an equivalent volume of saline solution. Systolic blood pressures of all rats were measured once per week, with the tail-cuff plethysmograph method using CODA™ noninvasive blood pressure system (Kent Scientific, Torrington, CT, USA).

### 2.4. Immunohistochemistry Analysis

Immunohistochemistry was used to detect the expressions of Collagen I, Collagen III, and Collagen IV. Nephridial tissues were fixed in 4% paraformaldehyde (pH = 7.4), embedded in paraffin, and sectioned at 4 *μ*m. Slides were incubated with rabbit polyclonal antibodies against Collagen I (1 : 400), Collagen III (1 : 600), and Collagen IV (1 : 1000). After washing with PBS, slides were incubated with biotinylated secondary antibody followed by conjugated HRP-labelled streptavidin (Maixing). Subsequently, slides were incubated with diaminobenzidine (Maixing) as the chromogen and counterstained with diluted Harris hematoxylin (Maixing). Slides were visualized under a light microscope (400x magnification), and six fields of view were randomly selected for each slide, and the average optical density of positive cells in each field was determined using true color multifunctional cell image analysis system (Image-Pro Plus, Media Cybernetics).

### 2.5. RNA Extraction and Real-Time PCR Analysis

RNA was extracted using TRIzol reagent (Takara Biotechnology Co., Ltd.) according to the manufacturer's instructions. RNA was reverse-transcribed to cDNA using PrimeScript II 1st strand cDNA Synthesis kit (Takara Biotechnology Co., Ltd.). Real-time PCR was performed using SYBR-Green premix (Applied Biosystems, Carlsbad, CA, USA) according to the manufacturer's instructions, under the following parameters: 40 cycles and 50°C for 2 min, 95°C for 7 min, 60°C for 15 s, and 60°C for 30 s. GAPDH was used as the internal control. PCR primer sequences (5′→ 3′) were shown in Table 1.

### 2.6. Western Blot Analysis

Total protein was extracted using RIPA lysis buffer containing protease and phosphatase inhibitor cocktails. BCA assay was used to determine the total protein concentrations, and equal amounts of proteins were resolved using 10% SDS-PAGE gels, transferred onto nitrocellulose membranes, and blocked. Membranes were incubated using primary antibodies for TGF-*β*1, Smad2/3, P-Smad2/3, Smad4, MMP-2, MMP-9, and GAPDH overnight at 4°C. Subsequently, membranes were incubated with the appropriate HRP-conjugated secondary antibodies and protein expressions were detected with enhanced chemiluminescence detection, using ChemiDoc XRS+ imaging system (Bio-Rad Laboratories, Inc., Hercules, CA, USA). Image Lab Software (Version 3.0) was used for densitometric analysis.

### 2.7. Statistical Analysis

All data were expressed as mean ± standard deviation (SD). One-way analysis of variance (ANOVA) was used to determine statistical significance, using SPSS software (version 18.0; SPSS, Inc., Chicago, IL, USA). *p* < 0.05 was considered as statistically significant.

## 3. Results

### 3.1. QXJYD Attenuated the Elevation of Blood Pressure in SHRs

**Figure 1:**
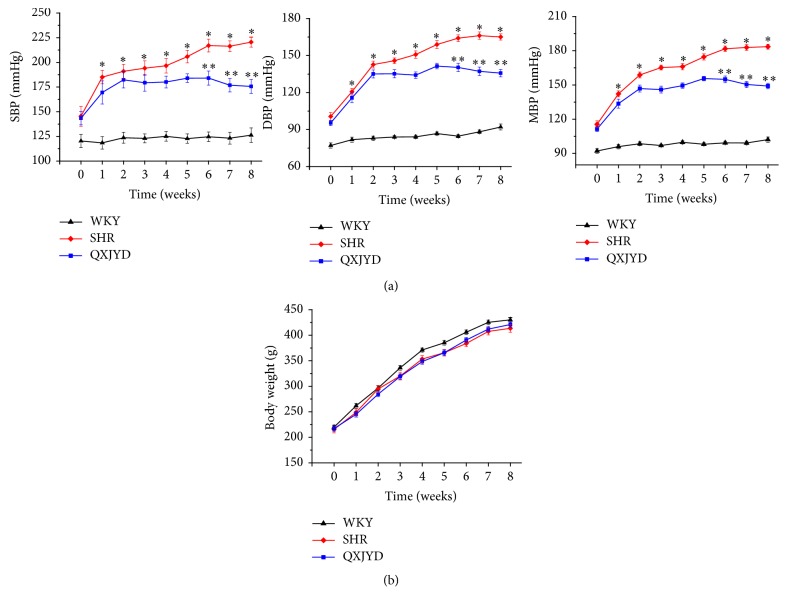
Effect of QXJYD treatment on blood pressure. (a) Systolic blood pressure (SBP), diastolic blood pressure (DBP), mean blood pressure (MBP), and (b) body weight were measured in spontaneously hypertensive rats (SHR, QXJYD) and Wistar Kyoto (WKY) rats (*n* = 10). All values were represented as mean ± SD. ^*∗*^*p* < 0.05, compared to WKY group; ^*∗∗*^*p* < 0.05, compared to SHR group.

We first measured the mean blood pressure (MBP) in SHRs to determine the effect of QXJYD on hypertension. Adverse effect of MBP was examined through determining body weight changes compared to normotensive control WKY rats. Treatment with QXJYD for five weeks significantly attenuated the elevation of MBP in SHRs (Figure 1(a)). Moreover, administration of QXJYD did not result in body weight changes in experimental rats, which demonstrated that QXJYD had no apparent toxicity (Figure 1(b)).

### 3.2. OXYJD Mitigated Glomerular Injury in SHRs

**Figure 2:**
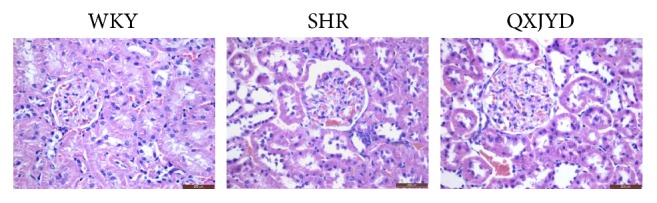
Effect of QXJYD treatment on mitigated damage of glomerulus. Histopathological changes of glomerulus in each group (*n* = 6) were observed by Harris Hematoxylin and Eosin (H&E) staining. Images were representatives taken at a magnification of 40x (top, scale bar = 200 *μ*m).

We next examined the histological changes in the kidneys of SHRs using Harris Hematoxylin and Eosin (H&E) staining. SHRs had significantly enlarged glomerulus and atrophy compared to the normotensive WKY rats and exhibited significant injuries to the tubulointerstitium including tubular dilation, disorder, and inflammatory cell infiltration (Figure 2). However, the injures to the glomerulus in SHRs were robustly mitigated by QXJYD treatment, which suggests that QXJYD can effectively relieve the damage to kidneys as a result of elevated blood pressure in SHRs.

### 3.3. QXJYD Decreased the Formation of Collagenous Fibers in SHRs

**Figure 3:**
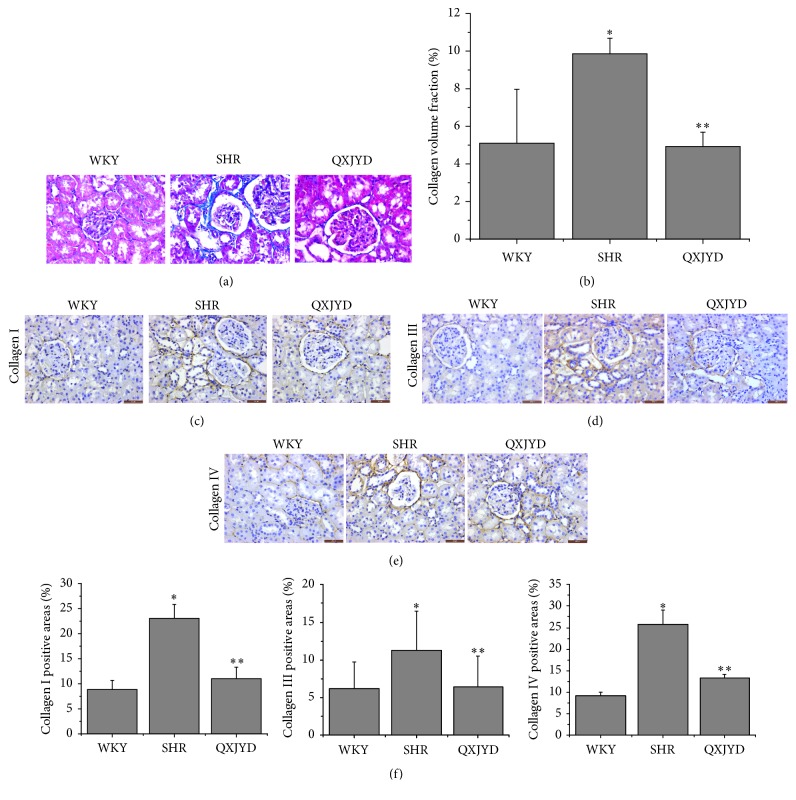
Effect of QXJYD treatment on the expression of collagen fiber in SHRs. (a) Representative image of Masson trichrome staining of kidney. (b) The Masson trichrome-positive tubulointerstitial area (blue) relative to the whole area from 6 random kidney fields was evaluated. Data are represented as the mean ± SEM (*n* = 6). Immunohistochemical analysis was performed to determine the protein expression of Collagen I (c), Collagen III (d), and Collagen IV (e) in renal interstitial from each group (*n* = 6). Images were representatives taken at a magnification of 40x. (f) Quantification of the mean expressions of Collagen I, Collagen III, and Collagen IV protein. All values were represented as the mean ± SD; ^*∗*^*p* < 0.05 compared to WKY group and ^*∗∗*^*p* < 0.05 compared to SHR group.

The degree of collagenous fiber formation in the kidneys of SHRs was measured using Masson's trichrome staining. There were extensive areas of collagen fiber deposition within the interstitial space in SHRs, which was reversed by QXJYD treatment (Figure 3(a)). Semiquantitative analysis of Masson's trichrome-positive areas revealed a significant increase in the formation of collagenous fibers in the SHRs compared to the normotensive WKY rats, which was significantly decreased following treatment with QXJYD (Figure 3(b)). To further examine the type of collagen fiber formation, we used immunohistochemical staining to analyze the expression of Collagen I, Collagen III, and Collagen IV in the renal interstitium. There was a significant increase in the expressions of Collagen I, Collagen III, and Collagen IV in SHRs, which was robustly attenuated following QXJYD treatment (Figures 3(c)–3(e)). Collectively, these data demonstrate that QXJYD can effectively attenuate the development of renal interstitial fibrosis during hypertension in SHRs.

### 3.4. QXJYD Protects against Renal Interstitial Injury via TGF-*β*/Smad Signaling Pathway

**Figure 4:**
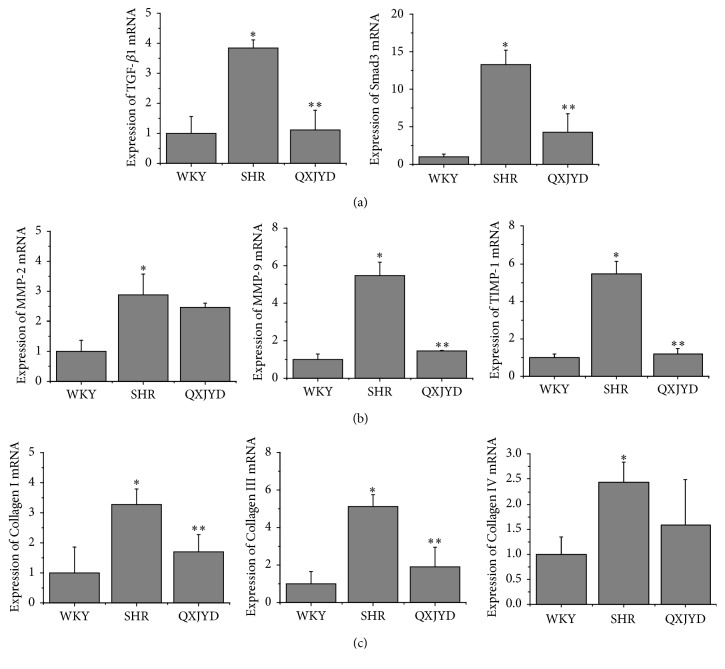
Effect of QXJYD treatment on the mRNA expression in SHRs. Q-PCR was performed to determine the mRNA expression of TGF-*β*1, Smad3 (a); MMP-2, MMP-9, and TIMP-1 (b); and Collagen I, Collagen III, and Collagen IV (c) in kidney issue from each group (*n* = 3). All values were represented as the mean ± SD; ^*∗*^*p* < 0.05 compared to WKY group and ^*∗∗*^*p* < 0.05 compared to SHR group.

The levels of protein and mRNA expression in SHRs were assessed using western blot and real-time PCR analyses. SHRs had significantly elevated levels of TGF-*β* and Smad3 mRNA expression compared to normotensive WKY rats, which was reversed following QXJYD treatment (Figure 4(a)). Moreover, SHRs had significantly higher mRNA expression of MMP-2, MMP-9, and TIMP-1 compared to normotensive WKY rats, which was also reversed following QXJYD treatment (Figure 4(b)). The mRNA expressions of Collagen I, Collagen III, and Collagen IV were similarly upregulated in SHRs and were reversed following QXJYD treatment (Figure 4(c)).

**Figure 5:**
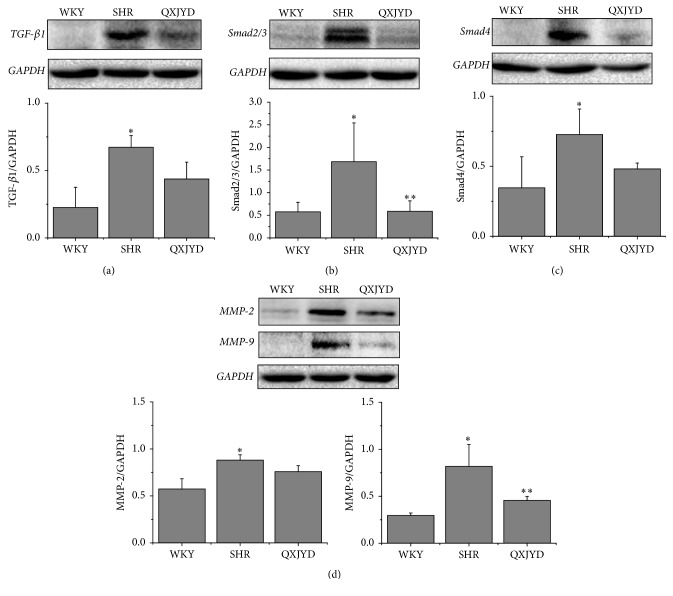
Effect of QXJYD treatment on the protein expression in SHRs. Western blot was performed to determine the protein expression of TGf-*β*1 (a), Smad2/3 (b), Smad4 (c), and MMP-2 and MMP-9 (d) in kidney issue from each group (*n* = 3). All values were represented as the mean ± SD; ^*∗*^*p* < 0.05 compared to WKY group and ^*∗∗*^*p* < 0.05 compared to SHR group.

In addition, SHRs had significantly increased protein expression levels of TGF-*β*1, Smad2/3, and Smad4 compared to normotensive WKY rats, which were attenuated following QXJYD treatment (Figures 5(a)–5(c)). Likewise, the protein expression levels of MMP-2 and MMP-9 were also significantly increased in SHRs and were attenuated following QXJYD treatment (Figure 5(d)). These data suggest that QXJYD reduced renal interstitial injury in SHRs via TGF-*β*/Smad signaling pathway.

## 4. Discussion

During the period of hypertension, reactive oxygen species (ROS), and lipid peroxidation products (MDA) have increased [17, 18]. At the same time, the body's antioxidant enzymes including superoxide dismutase (SOD), glutathione peroxidase (gsh-px), thioredoxin peroxidase (Prx), and catalase (CAT) activity were reduced [19–22]. Oxidative stress reaction in the kidney inherent cells, so as to activate a variety of transcription factors such as the NF-*κ*B to raise cytokine expression of TGF -*β*1 [23, 24]. There have been a number of studies confirm that the biological effect of TGF-*β*1 promote renal fibrosis could activate multiple signaling pathways, such as TGF-*β*1/Smads [25, 26].

This study demonstrated the therapeutic efficacy of QXJYD against hypertension, by suppressing the elevation of SBP using the SHR model. In addition, QXJYD showed no apparent toxicity and had no effect on body weight. To investigate the protective mechanism of QXJYD, we evaluated its effect on renal fibrosis, which is a pathological process associated with the progression of hypertension [27, 28]. QXJYD treatment significantly attenuated renal interstitial fibrosis in the SHR model, suggesting its protective effect in reducing renal damage. Excess accumulation of extracellular matrix in the glomerulus and renal interstitium was previously shown to be the main pathologic change associated with renal fibrosis [5, 29]. Our present study showed that QXJYD treatment could inhibit extracellular matrix (ECM) accumulation and thereby mitigate renal interstitial fibrosis in the SHR model.

TGF-*β*1 is a pleiotropic fibrosis-inducing cytokine involved in the development of renal interstitial fibrosis [30–33]. Previous research had suggested that TGF-*β*1 affected the synthesis of extracellular matrix and decreased the activation of MMPs [34]. Collagen is a major component of the extracellular matrix, while MMPs play important roles in the degradation of extracellular matrix [35]. Here we demonstrated that increased expressions of Collagens I, III, and IV proteins in the renal tissues of SHR model were associated with the accumulation of extracellular matrix. Notably, QXJYD treatment significantly attenuated the increased expressions of Collagens I, III, and IV proteins in SHR model. Moreover, QXJYD treatment also significantly decreased the elevated expressions of MMP-2 and MMP-9 proteins. This study demonstrated that QXJYD could attenuate excess extracellular matrix accumulation by inhibiting Collagens I, III, and IV expressions and increase the degradation of extracellular matrix by decreasing MMP-2 and MMP-9 expressions, thereby protecting against renal interstitial fibrosis in the SHR model.

Smad-dependent signaling pathway is recognized as one of the most classic signaling pathways involved in TGF-*β*1-induced fibrosis [36–38]. TGF-*β*1/Smad signaling pathway was demonstrated to be crucial in the regulation of renal interstitial fibrosis [25, 26]. Here we observed significantly increased expressions of Smad2/3 and P-Smad2/3 in renal tissues using the SHR model, which was reversed following treatment with QXJYD.

In summary, QXJYD mitigated the excess accumulation of extracellular matrix via regulating TGF-*β*1/Smad signaling pathway in the SHR model. In addition, QXJYD treatment significantly decreased the expressions of Collagens I, III, and IV, as well as the expressions of MMP-2 and MMP-9.

## 5. Conclusions

In the present study, we showed that the QXJYD could significantly attenuate the elevation of systolic blood pressure in an SHR model but had no effect on body weight changes, demonstrating its therapeutic efficacy against hypertension with no apparent toxicity. QXJYD treatment also significantly decreased the accumulation of extracellular matrix and mitigated renal interstitial fibrosis during hypertension, via regulation of TGF-*β*1/Smad signaling pathway.

## Figures and Tables

**Table 1:** The primer sequences of quantitative PCR analysis.

Gene name	Sequence
MMP2	F: GCCTCATACACAGCGTCAATCTT
	R: CGGTTTATTTGGCGGACAGT
MMP-9	F: CGTGTCTGGAGATTCGACTTGA
	R: TGGAAGATCGTGTGAGTTCC
TIMP-1	F: TCTGGCATCCTCTTGTTGCT
	R: CACAGCCAGCACTATAGGTCTT
Collagen I	F: GGCCAAGAAGACATCCCTGA
	R: CGTGCCATTGTGGCAGATAC
Collagen III	F: TGCAATGTGGGACCTGGTTT
	R: GGGCAGTCTAGTGGCTCATC
Collagen IV	F: GATCTCTAGAATGCAAGTGCGTGGAGTGTGCC
	R: GATCGCGGCCGCTTATGTCCTCTTCATGCATACT

## References

[1] World Health Organization (2013). *Global health risks: mortality and burden of disease attributable to selected major risks*.

[2] Liu Y. (2006). Renal fibrosis: new insights into the pathogenesis and therapeutics. *Kidney International*.

[3] Eddy A. A. (2005). Progression in chronic kidney disease. *Advances in Chronic Kidney Disease*.

[4] Strutz F., Zeisberg M. (2006). Renal fibroblasts and myofibroblasts in chronic kidney disease. *Journal of the American Society of Nephrology*.

[5] Hori Y., Katoh T., Hirakata M. (1998). Anti-latent TGF-*β* binding protein-1 antibody or synthetic oligopeptides inhibit extracellular matrix expression induced by stretch in cultured rat mesangial cells. *Kidney International*.

[6] Baricos W. H., Cortez S. L., Deboisblanc M. (1999). Transforming growth factor-beta is a potent inhibitor of extracellular matrix degradation by cultured human mesangial cells. *Journal of the American Society of Nephrology*.

[7] Feger M., Alesutan I., Castor T. (2015). Inhibitory effect of NH4Cl treatment on renal Tgf*β*1 signaling following unilateral ureteral obstruction. *Cellular Physiology and Biochemistry*.

[8] Böttinger E. P., Bitzer M. (2002). TGF-*β* signaling in renal disease. *Journal of the American Society of Nephrology*.

[9] Massagué J. (2012). TGF *β* signalling in context. *Nature Reviews Molecular Cell Biology*.

[10] Feng X.-H., Derynck R. (2005). Specificity and versatility in TGF-*β* signaling through smads. *Annual Review of Cell and Developmental Biology*.

[11] Derynck R., Zhang Y. E. (2003). Smad-dependent and Smad-independent pathways in TGF-*β* family signalling. *Nature*.

[12] Guo H., Zhang X., Cui Y. (2014). Isorhynchophylline protects against pulmonary arterial hypertension and suppresses PASMCs proliferation. *Biochemical and Biophysical Research Communications*.

[13] Tsai D.-S., Chang Y.-S., Li T.-C., Peng W.-H. (2014). Prescription pattern of Chinese herbal products for hypertension in Taiwan: A population-based study. *Journal of Ethnopharmacology*.

[14] Luan Y., Chao S., Ju Z.-Y. (2015). Therapeutic effects of baicalin on monocrotaline-induced pulmonary arterial hypertension by inhibiting inflammatory response. *International Immunopharmacology*.

[15] Liu P., Yan S., Chen M. (2015). Effects of baicalin on collagen I and collagen III expression in pulmonary arteries of rats with hypoxic pulmonary hypertension. *International Journal of Molecular Medicine*.

[16] Xiao F., He F., Chen H. (2016). Qingxuan Jiangya decoction reverses vascular remodeling by inducing vascular smooth muscle cell apoptosis in spontaneously hypertensive rats. *Molecules*.

[17] Reddy V. P., Zhu X., Perry G., Smith M. A., Bierhaus A. (2009). Oxidative stress in diabetes and alzheimer's disease. *Journal of Alzheimer's Disease*.

[18] Senador D., Key M., Brosnihan K. B. (2010). Cardiovascular interactions between losartan and fructose in mice. *Journal of Cardiovascular Pharmacology and Therapeutics*.

[19] Hou X., Shen Y. H., Li C. (2010). PPAR*α* agonist fenofibrate protects the kidney from hypertensive injury in spontaneously hypertensive rats via inhibition of oxidative stress and MAPK activity. *Biochemical and Biophysical Research Communications*.

[20] Zhao Q., Hixson J. E., Rao D. C. (2010). Genetic variants in the apelin system and blood pressure responses to dietary sodium interventions: A family-based association study. *Journal of Hypertension*.

[21] Li J.-M., Mullen A. M., Yun S. (2002). Essential role of the NADPH oxidase subunit p47phox in endothelial cell superoxide production in response to phorbol ester and tumor necrosis factor-*α*. *Circulation Research*.

[22] Lin F., Ma F., Chen C. (2013). Indapamide lowers blood pressure by increasing production of epoxyeicosatrienoic acids in the kidney. *Molecular Pharmacology*.

[23] Guyjarro C., Egido J. (2001). Transcription factor-kappa B (NF-kappa B) and renal disease. *Kidney International*.

[24] Lavoie P., Robitaille G., Agaharazii M., Lebel M., Lariviere R. (2004). Involvement of transforming growth factor-beta in the pathogenesis of hypertension in rats with chronic renal failure. *Journal of Hypertension*.

[25] Wang W., Koka V., Lan H. Y. (2005). Transforming growth factor-*β* and Smad signalling in kidney diseases. *Nephrology*.

[26] Lan H. Y. (2003). Tubular epithelial-myofibroblast transdifferentiation mechanisms in proximal tubule cells. *Current Opinion in Nephrology and Hypertension*.

[27] Hillege H. L., Girbes A. R. J., De Kam P. J. (2000). Renal function, neurohormonal activation, and survival in patients with chronic heart failure. *Circulation*.

[28] Laurent S., Boutouyrie P. (2015). The Structural Factor of Hypertension: Large and Small Artery Alterations. *Circulation Research*.

[29] Hosoo S., Koyama M., Kato M. (2015). The restorative effects of Eucommia ulmoides oliver leaf extract on vascular function in spontaneously hypertensive rats. *Molecules*.

[30] Baricos W. H., Cortez S. L., Deboisblanc M. (1999). Transform in growth factor *β*1 is apotent inhibitor of extracellula rmatrix degradation by cultured human mesangial cells. *Journal of the American Society of Nephrology*.

[31] Han W.-Q., Zhu Q., Hu J., Li P.-L., Zhang F., Li N. (2013). Hypoxia-inducible factor prolyl-hydroxylase-2 mediates transforming growth factor beta 1-induced epithelial-mesenchymal transition in renal tubular cells. *Biochimica et Biophysica Acta (BBA) - Molecular Cell Research*.

[32] Chang J., Wang H., Wang X. (2015). Molecular mechanisms of Polyphyllin I-induced apoptosis and reversal of the epithelial-mesenchymal transition in human osteosarcoma cells. *Journal of Ethnopharmacology*.

[33] Lamouille S., Xu J., Derynck R. (2014). Molecular mechanisms of epithelial-mesenchymal transition. *Nature Reviews Molecular Cell Biology*.

[34] García-Sánchez O., Sancho-Martínez S. M., López-Novoa J. M., López-Hernández F. J. (2015). Activation of the ALK-5 pathway is not per se sufficient for the antiproliferative effect of TGF-*β*1 on renal tubule epithelial cells. *Cellular Physiology and Biochemistry*.

[35] Kutz S. M., Hordines J., McKeown-Longo P. J., Higgins P. J. (2001). TGF-*β*1-induced PAI-1 gene expression requires MEK activity and cell-to-substrate adhesion. *Journal of Cell Science*.

[36] Wei M.-G., Sun W., Xiong P.-H., Shao J.-D. (2012). Antifibrotic effect of the Chinese herbs Modified Danggui Buxue Decoction on adriamycin-induced nephropathy in rats. *Chinese Journal of Integrative Medicine*.

[37] Overstreet J. M., Samarakoon R., Meldrum K. K., Higgins P. J. (2014). Redox control of p53 in the transcriptional regulation of TGF-*β*1 target genes through SMAD cooperativity. *Cellular Signalling*.

[38] Samarakoon R., Overstreet J. M., Higgins P. J. (2013). TGF-beta signaling in tissue fibrosis: redox controls, target genes and therapeutic opportunities. *Cellular Signalling*.

